# Optimized Design of the SGA-WZ Strapdown Airborne Gravimeter Temperature Control System

**DOI:** 10.3390/s151229781

**Published:** 2015-12-01

**Authors:** Juliang Cao, Minghao Wang, Shaokun Cai, Kaidong Zhang, Danni Cong, Meiping Wu

**Affiliations:** College of Mechatronics and Automation, National University of Defense Technology, Changsha 410072, China; 13755131212@163.com (J.C.); csk527@163.com (S.C.); kdzhang@263.net (K.Z.); hennessydlut@163.com (D.C.); meipingwu@263.net (M.W.)

**Keywords:** airborne gravimetry, SGA-WZ-02, optimized design, temperature control, heat analysis

## Abstract

The temperature control system is one of the most important subsystems of the strapdown airborne gravimeter. Because the quartz flexible accelerometer based on springy support technology is the core sensor in the strapdown airborne gravimeter and the magnet steel in the electromagnetic force equilibrium circuits of the quartz flexible accelerometer is greatly affected by temperature, in order to guarantee the temperature control precision and minimize the effect of temperature on the gravimeter, the SGA-WZ temperature control system adopts a three-level control method. Based on the design experience of the SGA-WZ-01, the SGA-WZ-02 temperature control system came out with a further optimized design. In 1st level temperature control, thermoelectric cooler is used to conquer temperature change caused by hot weather. The experiments show that the optimized stability of 1st level temperature control is about 0.1 °C and the max cool down capability is about 10 °C. The temperature field is analyzed in the 2nd and 3rd level temperature control using the finite element analysis software ANSYS. The 2nd and 3rd level temperature control optimization scheme is based on the foundation of heat analysis. The experimental results show that static accuracy of SGA-WZ-02 reaches 0.21 mGal/24 h, with internal accuracy being 0.743 mGal/4.8 km and external accuracy being 0.37 mGal/4.8 km compared with the result of the GT-2A, whose internal precision is superior to 1 mGal/4.8 km and all of them are better than those in SGA-WZ-01.

## 1. Introduction

The information about the Earth’s gravity field is becoming more and more important in geophysics, geodesy, geodynamics, *etc.* Airborne gravimetry is an effective method to collect highly precise information about large areas of the gravity field. Different principles are used in different airborne gravimetry systems; the three-axis inertial platform navigation system is used in GT-1A, GT-2A and AIRGrav, the 2-axis stable platform technique is used in LCR Air/Sea Gravity System, and Strapdown Inertial Navigation System (SINS) is used in SISG, GT-X and SGA-WZ [1].

The strapdown airborne gravimeter has the advantages of being structurally simple, small sized, light weight, low cost and low power consumption. The strapdown gravimeter is also able to implement vector gravimetry, and gravity vector information is significant in many subjects, like geophysics [2,3]. Therefore, airborne gravimeters based on SINS have been a major development trend in airborne gravimetry.

The core sensor in the strapdown airborne gravimeter (e.g., SGA-WZ) is the quartz flexible accelerometer. The advantages of the quartz flexible accelerometer are good stability and simple structure, but the output of the quartz flexible accelerometer has an obvious drift due to temperature variation because the quartz flexible accelerometer is based on springy support technology and the magnet steel in electromagnetic force equilibrium circuits of quartz flexible accelerometer is greatly affected by temperature. Under general inertial navigation conditions, the problem of temperature drift can be solved through the method of temperature compensation [4]. However, at the mGal (10^−5^ m/s^2^) level of high precision airborne gravimetry applications, temperature compensation cannot meet the demands of airborne gravimetry. In most mature airborne gravimetry systems, temperature control measurements are adopted. The temperature control precision in the GT-2A system is better than 0.1 °C, and its day drift is smaller than 0.1 mGal. The temperature control precision in the AIRGrav system is better than 0.05 °C. Some airborne gravimeter systems cannot provide practical results, because their thermal control accuracy cannot meet the requirements. Addressing the problem of airborne gravimeter temperature control systems, some research, according to the structure and material properties of the quartz flexible accelerometer, have built the temperature field distribution of the quartz flexible accelerometer under the condition of only considering heat transfer [5]. Others, mainly focusing on control algorithm, have ignored the importance of the thermal structure design [6,7,8]. What’s more, research about airborne gravimeter temperature control systems is rare. Based on the design experience of the SGA-WZ-01, the first strapdown airborne gravimeter developed in China, the temperature control system and the thermal structure are optimized redesigned in the SGA-WZ-02.

In the following section, the temperature control system in SGA-WZ-01 is presented and its temperature control system shortcomings are pointed out. In the Section 3, the optimized design of the SGA-WZ-02 temperature control system is described. The experimental results are given in Section 4. Finally, a discussion and some conclusions are given in Section 5.

## 2. Description of the Temperature Control System in SGA-WZ-01

### 2.1. The Three-Level Temperature Control System in SGA-WZ-01

The temperature drift of the quartz flexible accelerometer used in the SGA-WZ is about 10 mGal/°C and the max permitted error caused by the quartz flexible accelerometer in the SGA-WZ is 0.5 mGal. To meet the precision requirements of the SGA-WZ airborne gravimeter, the temperature stability in the SGA-WZ should be superior to 0.05 °C [9]. To achieve this precision, the temperature control system in the SGA-WZ should be carefully designed. The alternative temperature control systems are a single-level temperature control system or a multi-level temperature control system. In practice, the highest precision of a single-level temperature control system is about 0.1 °C, which can’t meet the system requirements.

**Figure 1:**
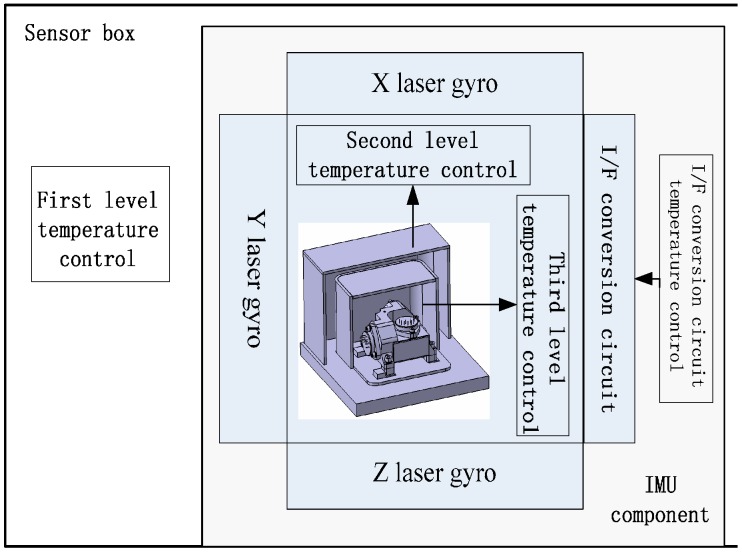
The sketch of the SGA-WZ temperature control system.

To ensure the temperature control precision and minimize the effect of temperature on the gravimeter, the SGA-WZ temperature control system therefore adopts a three-level modular control method. A sketch of the SGA-WZ temperature control system is shown in Figure 1.

Every level of the temperature control unit has its own thermal insulation layer, heating element, temperature sensors, drive module, *etc.* The first level temperature control aims at keeping the temperature of the sensor box as homogeneous and stable as possible, providing a good working condition to lase gyros and a preliminary isolation of the effect of temperature on the quartz flexible accelerometer. The second level temperature control tries to manage the air temperature of the accelerometer component and further isolates the effect of temperature on the quartz flexible accelerometer. On the basis of the former two level control units, the third level temperature control directly controls the temperature of the quartz flexible accelerometer.

### 2.2. The Performance of SGA-WZ-01 Temperature Control System

**Figure 2:**
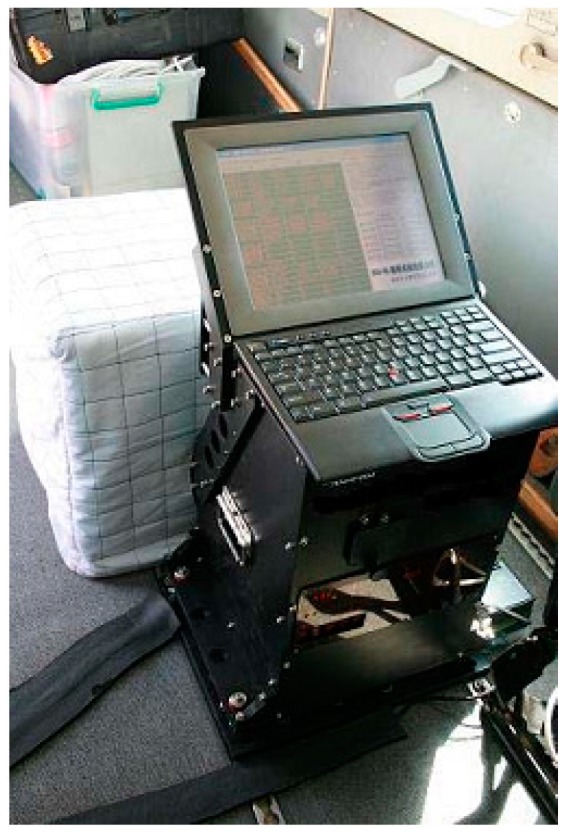
The photo of SGA-WZ-01.

The SGA-WZ-01 has already undergone 2400 h of static tests and about 2000 km surveying operation, with the precision of the SGA-WZ reaching 1.5 mGal/4.8 km. The tests and surveying results show that the temperature control system in SGA-WZ can essential satisfy the requirements. Figure 2 is a photo of the SGA-WZ-01, where the electric box is in the front of the picture and the sensors box is behind the electric box (the white one). 

**Figure 3:**
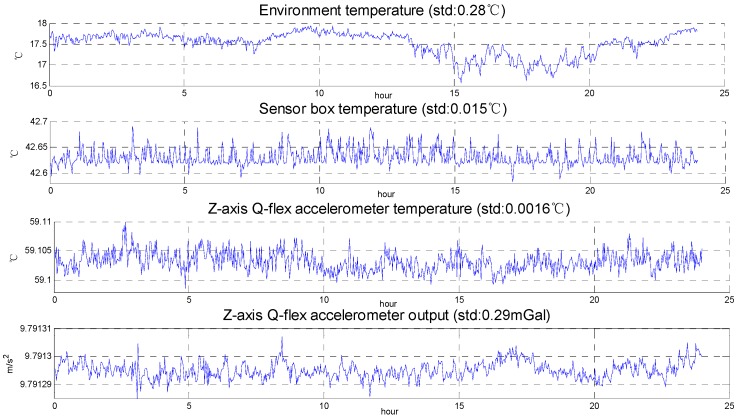
Temperature change and Z-axis accelerometer output of static tests.

**Figure 4:**
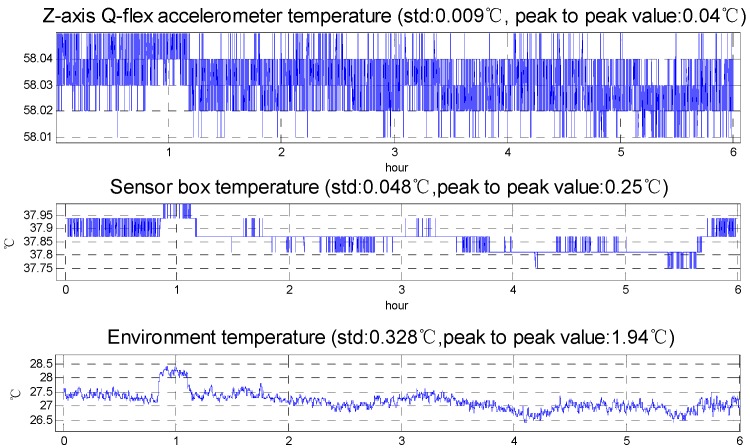
Temperature change in surveying and mapping operation.

**Figure 5:**
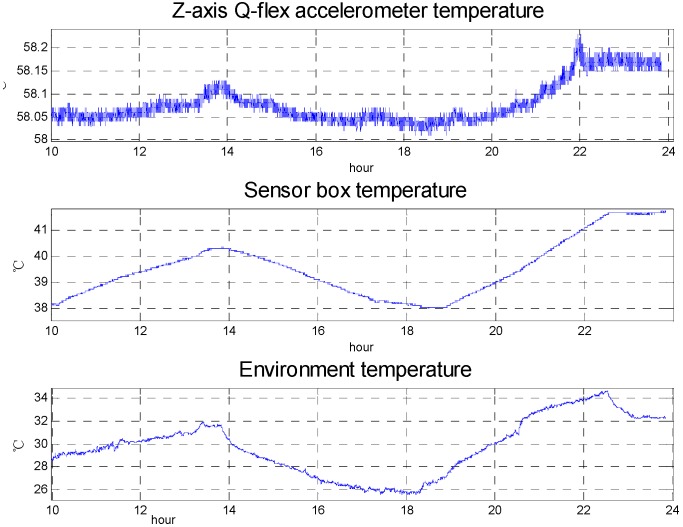
The performance of temperature control system in hot weather.

Figure 3 shows the temperature change and quartz flexible accelerometer output in the static test, while Figure 4 shows the temperature change in the surveying and mapping operation and represents that the temperature control system meets the requirement and the effects of temperature on the SGA-WZ are below the index in the surveying and mapping operations. The SGA-WZ-01 temperature control system only uses heating to keep the temperature stable, that is to say, the natural heat dissipation is the only way of cooling. In this situation, the set value of the 1st level temperature control system must be higher than the environmental temperature by about 10 °C. With large numbers of flight experiments, the control method using only heating has the following shortcomings:

(1) The aircraft cabin easily exceeds the set value of the 1st level temperature control unit in hot weather. As a result of a lack of heat dissipation, the temperature can’t meet the stability requirements and further impacts the quartz flexible accelerometer’s precision. Figure 5 shows the performance of the temperature control system in hot weather.
(2) Laser gyro’s best working temperature is at about 30 °C, but the set value of the 1st level temperature control unit is set at 40 °C. Higher temperatures affect the service life of the laser gyro and reduce the reliability of the system.
(3) Figure 1 and Figure 2 show that the sensors’ box is so big that it increases the power consumption of the temperature control system by some degrees.
(4) The SGA-WA-01 design is too conservative to make the SGA-WA-01 relatively light, for there have not been any previous experiments.

## 3. SGA-WZ-02 Temperature Control System Optimized Design

**Figure 6:**
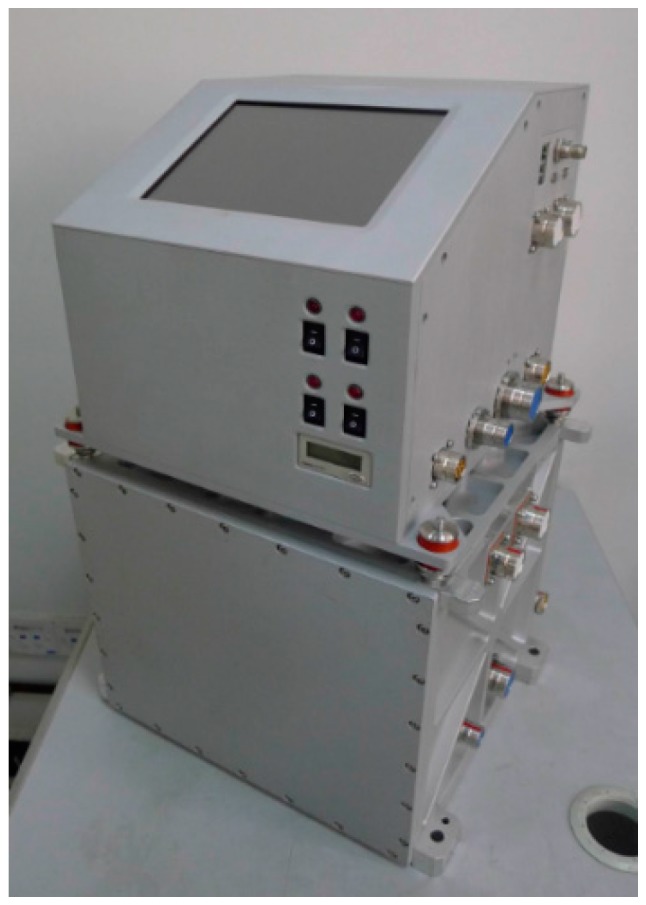
Photo of the SGA-WZ-02.

The SGA-WZ-02 is the new airborne gravimeter developed by the Laboratory of Inertial Technology of the National University of Defense Technology. The airborne experiments show that the precision of SGA-WZ-02 is better than 1 mGal/4.8 km. Figure 6 shows a photo of the SGA-WZ-02, where the sensor box is on the bottom of the electric box.

### 3.1. The Optimized 1st Level Temperature Control Design Based on a Thermoelectric Cooler

In order to conquer the problem caused by hot weather in the SGA-WZ-01 Temperature Control System, a thermoelectric cooler (TEC) is adopted in the 1st level temperature control. TEC is based on the Peltier Effect, which is the presence of heating or cooling at an electrified junction of two different conductors. Therefore, the TEC works like a solid-state active heat pump which transfers heat from one side of the device to the other, with some consumption of electrical energy.

**Figure 7:**
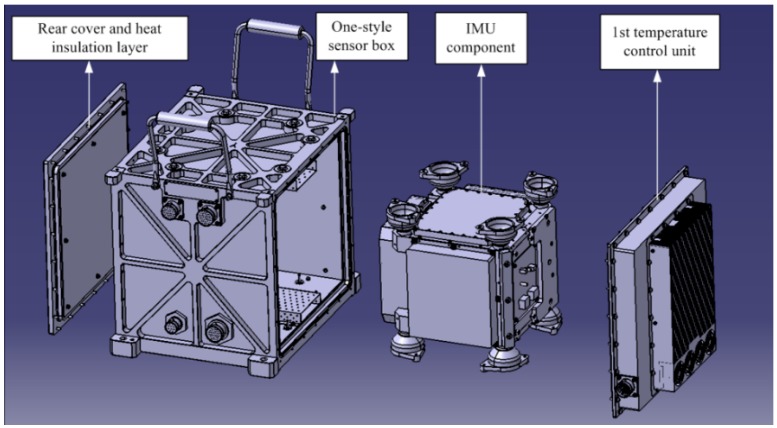
The structure of the 1st level temperature control in SGA-WZ-02.

**Figure 8:**
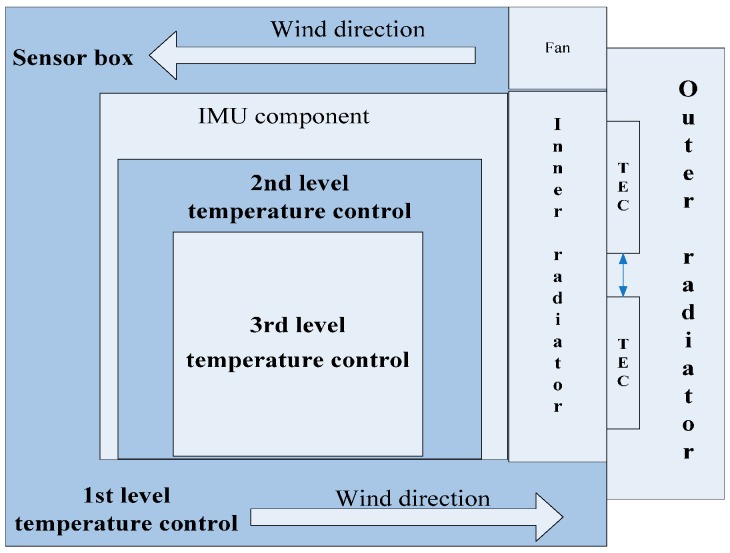
Sketch of the 1st level temperature control in SGA-WZ-02.

The temperature control system of the SGA-WZ-02 adopts a one-style design with compact and reasonable structure, prominently reducing the volume and the power consumption. The structure of the 1st level temperature control is shown in Figure 7 and a sketch of the optimized 1st level temperature control design is shown in Figure 8.

**Figure 9:**
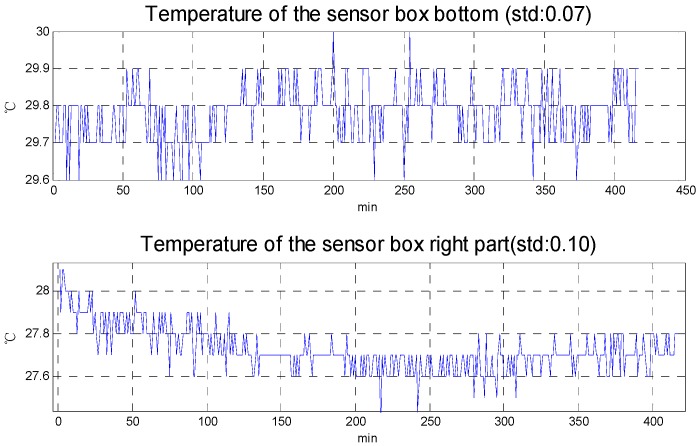
The stability experiment results.

In order to estimate the performance of the new design, stability experiments and max cool down capability experiments are carried out. Figure 9 shows the result of the stability experiments. In the stability experiments, the set temperature is 30 °C. Figure 9 indicates that a temperature gradient exists in the temperature control system, but the temperature gradient is unavoidable in every single input temperature control system. The decisive element in the temperature control system of the airborne gravimeter is its stability, and Figure 9 shows that the temperature stability meets the system requirements (superior to 0.5 °C).

**Figure 10:**
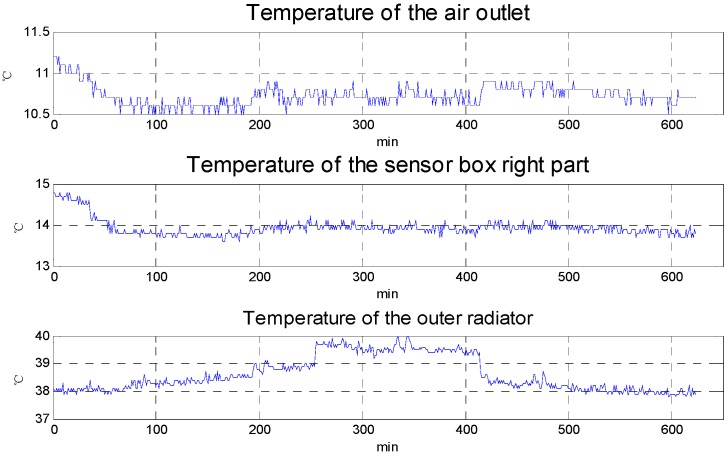
The max cool down capability experiment results.

Figure 10 shows the results of the max cool down capability experiments. Room temperature is about 25 °C in the experiments. To evaluate the cool down capability, the set temperature is 0 °C. The temperature difference between the outer radiator and the air outlet is about 30 °C, so that the temperature difference essentially reaches the max temperature difference of the selected TEC and proves the 1st level temperature control unit has a good heat sink capability. From the experimental results, the max cool down capability is about 15 °C compared with the air outlet temperature and about 11 °C compared with the right part of sensor box. Figure 5 shows that the max temperature rise in the aircraft cabin is less than 10 °C, so the cool down capability of 1st level temperature control meets the requirements for actual use.

### 3.2. The 2nd and 3rd Level Temperature Control Unit Heat Analysis

As a result of the equipment being a newly optimized design, heat analysis is needed in the 2nd and 3rd level temperature control units to meet the temperature control precision requirement. In the 2nd and 3rd level temperature control units, three basic ways of heat transfer (radiation, conduction and convection) exist to different degrees. The main forms of heat exchange in temperature control units can be any of the following three:

(1) Heat conduction of the component itself;
(2) The radiation from the unit surfaces to the environment;
(3) The convection between the internal and external surface of unit heat transfer.

Besides the ways of heat exchange, the material’s thermal physical properties, boundary conditions, related thermal parameters and the heat transfer coefficient used in the calculations are necessarily clarified in the heat analysis. The thermal physical properties are listed in Table 1.

**Table 1:** Material physical properties.

Material	Density $\left(\mathrm{kg}\cdot {\text{m}}^{3}\right)$	Specific Heat (J/(kg·k))	Heat Conduct Coefficient $\left(\text{W}/({\text{m}}^{2}\cdot \text{k})\right)$
Steel	7840	450	49.8
Heat insulation material	30	1400	0.03

**Figure 11:**
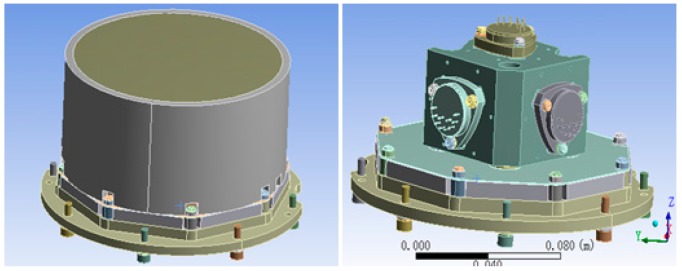
Structure diagram of the accelerometer installation with temperature control system.

The quartz flexible accelerometer unit, which contains 2nd and 3rd level temperature control units, is shown in Figure 11. The quartz flexible accelerometer unit is assembled into the IMU component from the bottom side. The 2nd level heating is on the outer wall of the unit shell. The heat insulation measures use foam in the outer of 2nd level heating sheet. The 3rd level heating is on the bottom of the accelerometer base and directly controls the temperature of the accelerometer.

**Figure 12:**
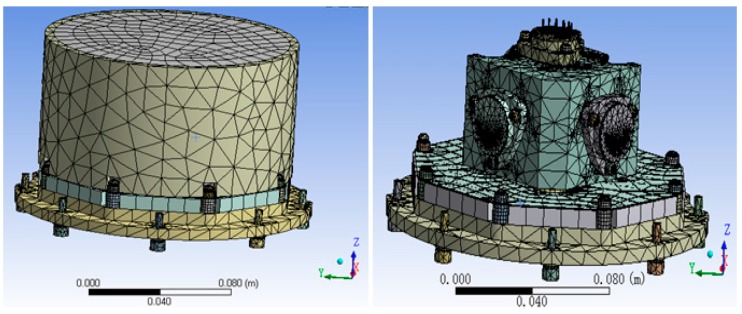
The finite element model of the accelerometer unit.

Using the finite element analysis software ANSYS [10] to analyze the temperature field of accelerometer unit, we can make an appropriate simplification. We ignore the accelerometer’s heat generation, because accelerometer’s heat generation is much less than that of the heat sheet. We also need to refine finite element mesh division of the structure joint, like heat transfer of the accelerometer fixed screw in the internal system [11]. Finally, as the finite element model of accelerometer unit in Figure 12 shows, the nodes and units of the division of finite elements are 258,755 and 111,539, respectively. Needless to say, intensive meshing is helpful to improve the accuracy of the simulation [12].

**Figure 13:**
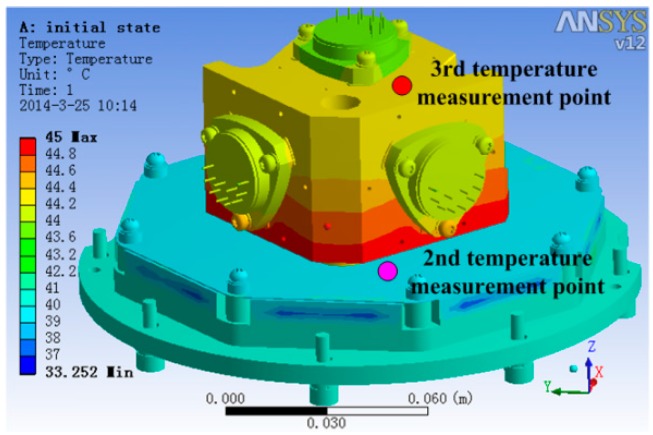
The steady-state temperature field of the accelerometer unit.

To decide the temperature measurement point in the 2nd and 3rd level temperature control units, ANSYS was used to analyze the temperature field. The temperature field simulation result is shown in Figure 13. In the analysis, the 2nd level heating temperature is set at 40 °C and that of 3rd level is 45 °C. From the simulation results, it is obvious that the temperature of accelerometer unit foundation bed’s top surface and the temperature of the accelerometer base’s top surface are relatively uniform. Thus, setting the 2nd level temperature control system temperature measurement point on the accelerometer unit foundation bed’s top surface and setting the 3rd level temperature control system temperature measurement point on the accelerometer base’s top surface is better than setting it on other places, because the single point represents the temperature of the whole surface on those surfaces and makes the control system’s input reflect the temperature change of the whole unit.

**Figure 14:**
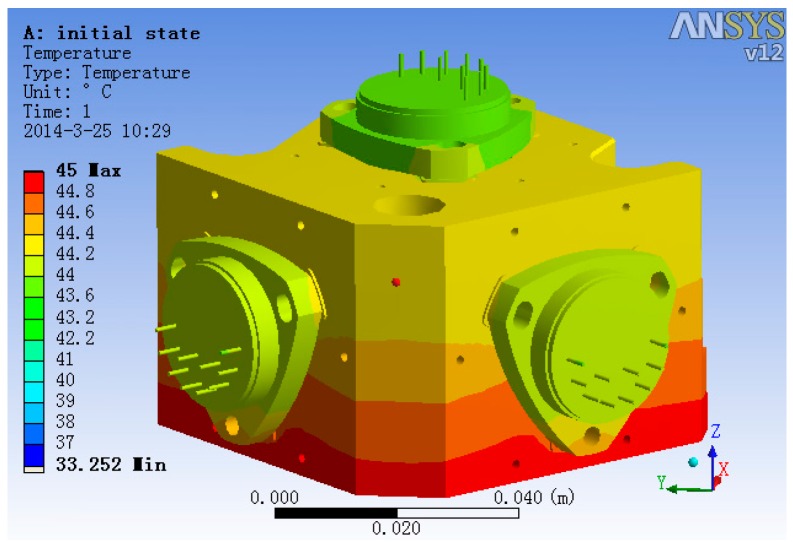
The steady-state temperature field of the accelerometer base.

Hiding all other structures and with heater open, the steady-state temperature field of accelerometer’s base and three accelerometers is shown in Figure 14. From Figure 14, we can get the information that the temperature gradient of the two lateral accelerometers is much larger than that of the plumb accelerometer, and the max temperature difference of the two lateral accelerometers head is 0.15 °C, while the max temperature difference of the plumb accelerometer head is 0.05 °C.

**Figure 15:**
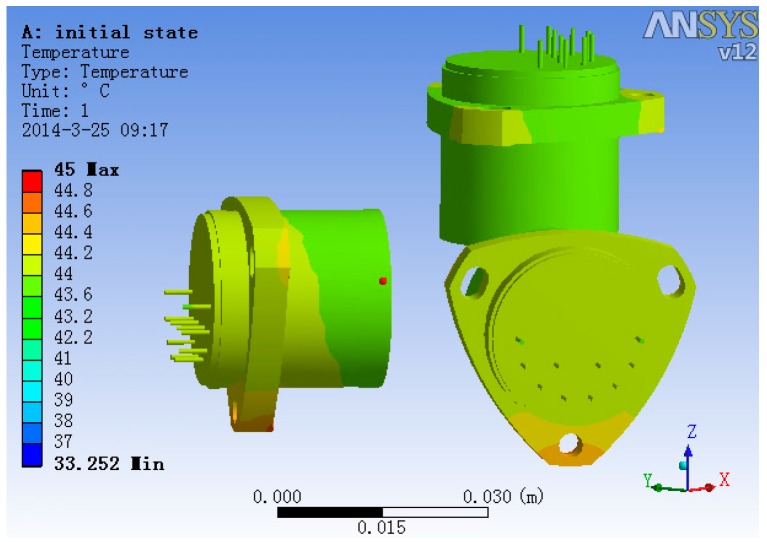
The temperature field of the three accelerometers.

The temperature field of the three accelerometers is shown in Figure 15. It is evident that the plumb accelerometer body’s temperature field is much more consistent than that of the two lateral accelerometers, of which the max temperature difference is 0.44 °C, while the plumb accelerometer body’s max temperature difference is 0.18 °C.

The analysis results show that the temperature control methods significantly improve the uniformity of the temperature field of the accelerometers compared with the accelerometer temperature data in general conditions [9], which benefits the stability of the accelerometer’s output.

### 3.3. The Optimized Design of the 2nd and 3rd Level Temperature Control

**Figure 16:**
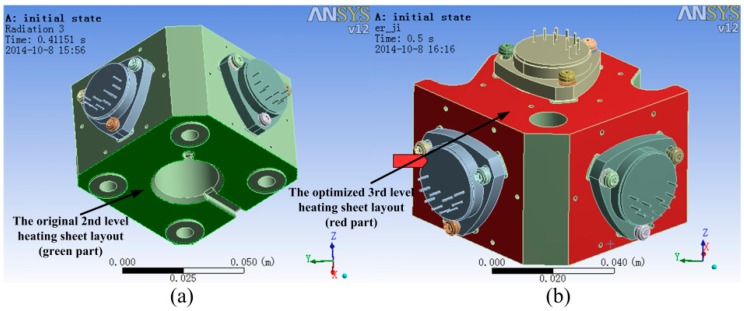
(**a**) The original deployment of 3rd level heating sheet layout (high light green part); (**b**) The optimized deployment of 3rd level heating sheet layout (high light red part).

**Figure 17:**
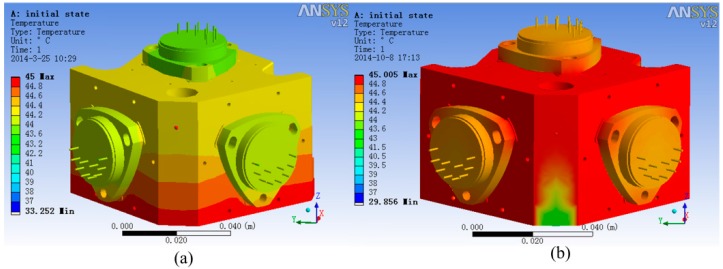
(**a**) The original temperature field; (**b**) The optimized temperature field.

In order to minimize the temperature gradient, we should optimize the layout of the heating sheet. The original 3rd level heating sheet is deployed on the bottom of the accelerometer base, as shown in Figure 16a. The optimized deployment of the second-level heat sheet is shown in Figure 16b. The temperature field comparison of the accelerometer unit using ANSYS, is shown in Figure 17. Comparing the temperature field in the original layout with the optimized one, the newly deployed scheme can significantly lessen the temperature difference in the two lateral accelerometers, so that the temperature field can be more homogeneous. The detailed datum of temperature differences in the two deployment scheme is shown in Table 2.

**Table 2:** Temperature differences in the two layout method.

Layout Method	Two Lateral Accelerometers Max Temperature Difference	Plumb Accelerometers Max Temperature Difference
The original	0.44 °C	0.18 °C
The optimized	0.21 °C	0.17 °C

**Figure 18:**
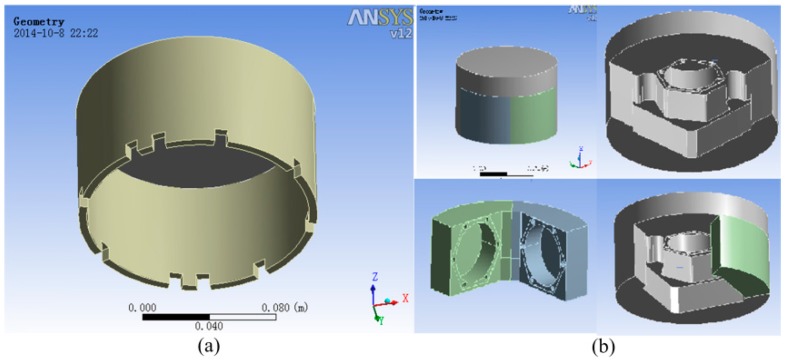
(**a**) The original heat insulating layer; (**b**) The optimized heat insulating layer.

Besides using a new heating sheet layout to reduce the temperature gradient of accelerometer unit, intensifying the heat insulation is another good method. The heat insulating layer currently used in the accelerometer unit is shown in Figure 18a. 

As seen in Figure 18, there is a large amount of air in the whole unit and the air’s heat transfer conditions are relatively complex, which can influence the temperature stability. The optimized heating layout is shown in Figure 18b and the material used in the two structures is the same (thermal conductivity ≤ 0.03 W/m·k). The mold with an optimized heat insulating structure and low coefficient of thermal coefficient coats the accelerometer unit. The new insulating structure reduces the amount of air in the unit and increases the thickness of insulating layer, so it is conducive to the temperature stability of the accelerometers.

## 4. The Performance of SGA-WZ-02 Airborne Gravimeter

**Figure 19:**
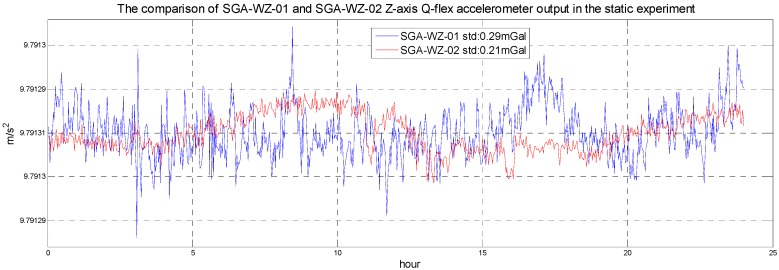
The comparison of SGA-WZ-01 and SGA-WZ-02 Z-axis quartz flexible accelerometer output in the static experiment.

In order to check the performance of SGA-WZ-02 with the optimized temperature control system design, both a static experiment and an airborne experiment are carried out. The output of SGA-WZ-02 Z-axis quartz flexible accelerometer in the static experiment is shown in Figure 19 and compared with that in SGA-WZ-01. Compared with Figure 3, the z-axis quartz flexible accelerometer output in the SGZ-WZ-02 is better than the accelerometer output in the SGZ-WZ-01 and the accuracy satisfies the needs of airborne gravimetry.

**Figure 20:**
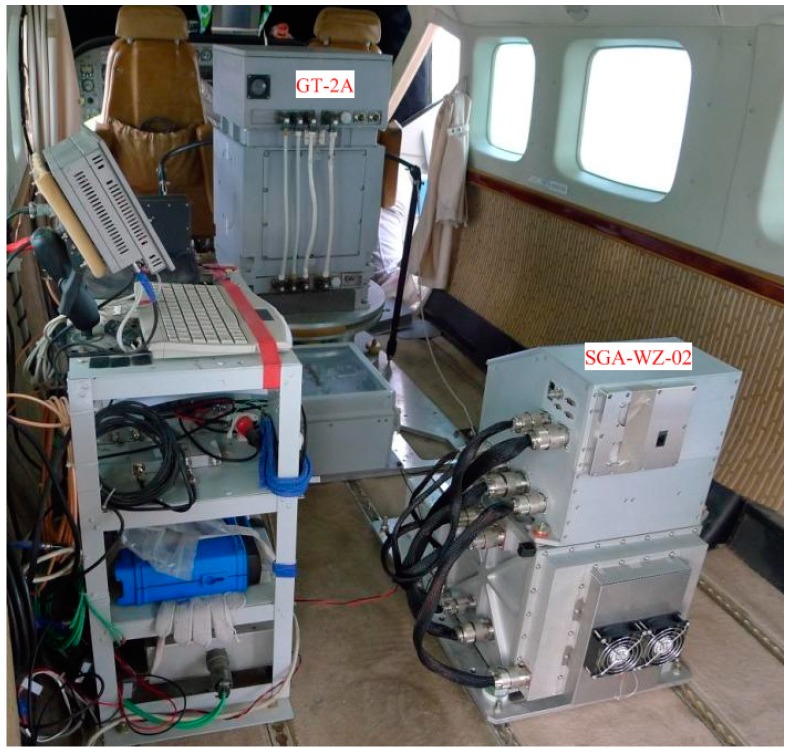
Cabin of the aircraft.

**Figure 21:**
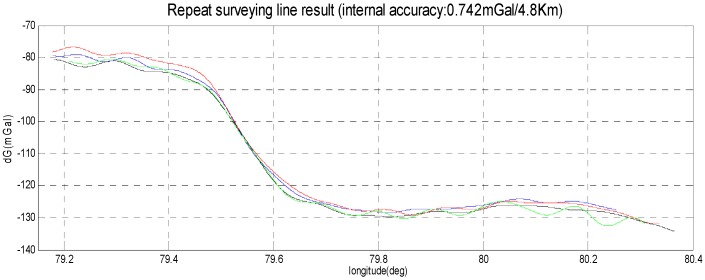
The repeat surveying line result (internal accuracy: 0.742 mGal/4.8 km).

**Figure 22:**
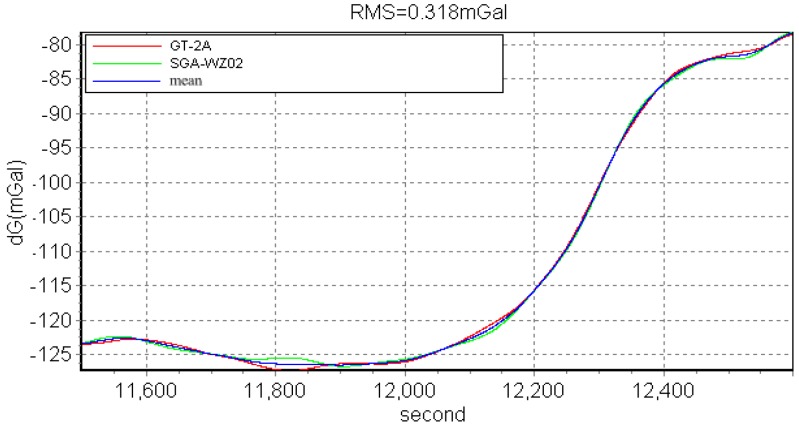
The surveying result compared with GT-2A.

**Figure 23:**
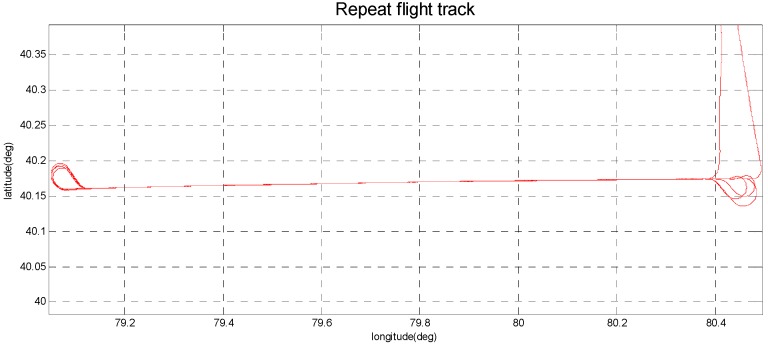
The flight track of repeat line experiment.

The SGA-WZ-02 airborne experiment was carried out in western China with GT-2A in the same aircraft, and Figure 20 shows the aircraft cabin, in which both SGA-WZ-02 and GT-2A are shown. The result of repeated surveying is shown in Figure 21 and the result comparison with GT-2A is shown in Figure 22, with the internal accuracy being 0.742 mGal/4.8 km in the experiment and the external accuracy compared with the result of GT-2A being 0.37 mGal. The flight track is shown in Figure 23. The result is better than the internal accuracy in SGA-WZ-01, which is about 1.5 mGal/4.8 km.

In the experiment, the problem of temperature control system seen in SGA-WZ-01 does not appear in SGA-WZ-02. The reason is that the TEC used in the 1st level temperature control works well enough to neutralize the temperature change caused by inner heat generation and hot weather, and the optimized design of the 2nd and 3rd level temperature control guarantees the stability of the quartz flexible accelerometer output.

## 5. Conclusions

The optimized design of the temperature control system in the SGA-WZ-02 based on the design experience of the SGA-WZ-01 system was presented. The SGA-WZ-01 cannot overcome the temperature changes caused by hot weather and the set value of the 1st level temperature control was too high to ensure the service life of laser gyros, so TEC is added to the temperature control system of the SGA-WZ-02. The experiments indicate that the stability accuracy of the new 1st level temperature control system is superior to 0.1 °C, and the max cool down capability is about 15 °C and 11 °C compared with the outlet air temperature and the right part of sensor box, respectively. Using ANSYS, a finite element model of the 2nd and 3rd level temperature control components is built to analyze the temperature field. Through heat analysis, besides the new temperature measurement points of the 2nd and 3rd level temperature control system selected, the optimized heating sheet layout and the new heat insulating layer are given. The max temperature difference of the accelerometer is 0.44 °C before the optimized design and 0.21 °C after optimizing the heating sheet layout. A new heat insulating layer is used to guarantee the temperature stability of the accelerometer. Both the static experiments (0.21 mGal/24 h) and airborne experiments (0.742 mGal/4.8 km) of SGA-WZ-02 show better results compared with those of SGA-WZ-01 (0.28 mGal/24 h in static experiments and 1.5 mGal/4.8 km in airborne experiments). The conclusion of the optimized design can provide meaningful ideas for the construction of the strapdown airborne gravimeter temperature control system, and can also offer references for the temperature compensation [13] and heat optimization of strapdown inertial navigation systems.
